# Differential response of rat strains to obesogenic diets underlines the importance of genetic makeup of an individual towards obesity

**DOI:** 10.1038/s41598-017-09149-6

**Published:** 2017-08-22

**Authors:** Muralidhar MN, Prasad SMVK, Kiran Kumar Battula, Giridharan NV, Rajender Rao Kalashikam

**Affiliations:** Laboratory of Molecular Genetics, National Centre for Laboratory Animal Sciences, National Institute of Nutrition, Hyderabad, 500 007 Telangana India

## Abstract

Obesity, a multifactorial disorder, results from a chronic imbalance of energy intake vs. expenditure. Apart from excessive consumption of high calorie diet, genetic predisposition also seems to be equally important for the development of obesity. However, the role of genetic predisposition in the etiology of obesity has not been clearly delineated. The present study addresses this problem by selecting three rat strains (WNIN, F-344, SD) with different genetic backgrounds and exposing them to high calorie diets. Rat strains were fed HF, HS, and HFS diets and assessed for physical, metabolic, biochemical, inflammatory responses, and mRNA expression. Under these conditions: significant increase in body weight, visceral adiposity, oxidative stress and systemic pro-inflammatory status; the hallmarks of central obesity were noticed only in WNIN. Further, they developed altered glucose and lipid homeostasis by exhibiting insulin resistance, impaired glucose tolerance, dyslipidemia and fatty liver condition. The present study demonstrates that WNIN is more prone to develop obesity and associated co-morbidities under high calorie environment. It thus underlines the cumulative role of genetics (nature)﻿ and diet (nurture) towards the development of obesity, which is critical for understanding this epidemic and devising new strategies to control and manage this modern malady.

## Introduction

Obesity is a multifactorial disorder characterized by excessive weight gain, due to a chronic imbalance of energy intake versus expenditure, with increased health problems leading to reduced life expectancy^1–5^. Increased consumption of dietary carbohydrates/fats is regarded as one of the most important environmental factors predisposing one to obesity in modern societies^6–8^. But the ability of dietary carbohydrates/fats to promote body mass gain need not exclusively be due to its caloric values, since prolonged caloric pair feeding seems to retain most of the obesogenic potential of different high carbohydrate/fat diets^9, 10^. It is well established that high fat (HF) diet promotes weight gain; aids in the progression towards impaired glucose tolerance (IGT), insulin resistance and chronic low-grade inflammation, which contributes to the pathogenesis of metabolic syndrome and other chronic diseases^11–13^. Studies have reported that apart from high calorie diet consumption, a certain amount of genetic predisposition is also equally important in the development of metabolic syndrome^14–16^. Indeed it has been proposed that interactions between obesity genotypes and an obesogenic environment would synergistically augment the frequency of obesity^17^. The extent of predisposition towards obesity in an obesogenic environment can vary among individuals and this variability may be mainly attributed to the genetic variation. Among the different mechanisms that could lead to inter individual differences in fat deposition and obesity, the epigenetic regulation of gene expression has emerged in the last few years as the most potential contributor^18, 19^. The interplay of various dietary and environmental factors influence epigenetic events; causing differential gene expression and inter-individual differences resulting in susceptibility to develop obesity and other metabolic diseases^20, 21^. Further, elucidating the mechanisms involved in the consumption of high calorie diet-induced obesity is of major importance to understand the pathophysiology of human obesity, since at present the diet pattern has shifted from natural foods to processed food, the later being loaded with high calories. Therefore, determining how allelic and environment variations interact to determine obesity phenotypes are critical for understanding the obesity epidemic.

Animal studies show that while some inbred strains of mice and rats are susceptible to obesity when fed on high fat diet, others are found to be resistant and these phenotypic differences could be due to diet-gene interaction^22–25^. Identifying suitable animal model to study the impact of genetic variation on predisposition of individual’s obesogenic tendency can greatly advance obesity research. The present study address this issue by carrying out investigation on three rat strains (WNIN, F-344, SD) having different genetic backgrounds^26^ by exposing them to a high calorie diet. Preliminary studies in these strains had shown that they differ from each other, in terms of percentage of fat per 100 g-body weight and in physical activity^27^. Further to mention, the two﻿ obese rat strains﻿ (WNIN/Ob and WNIN/GR-Ob) evolved spontaneously from WNIN strain (established in NIN), the former with euglycemia and the latter with impaired glucose tolerance (IGT) respectively^28^. Essentially, the idea was thus to test the obesogenic tendency of these strains, which differed from each other physically, biochemically and genetically by exposing them to high calorie diet and see their ability to switch on/off genes associated with obesity and their likely epigenetic regulation. We hope, such an approach would answer the initial query as to why some individuals are more susceptible than others to (diet- induced) obesity when exposed to the same nutritional environment. It should also give us new insights into the interplay between nature and nurture in the prevention of metabolic syndrome and hopefully open up new avenues for its control and management.

## Results

### Nutritional measurements and body composition

**Figure 1:**
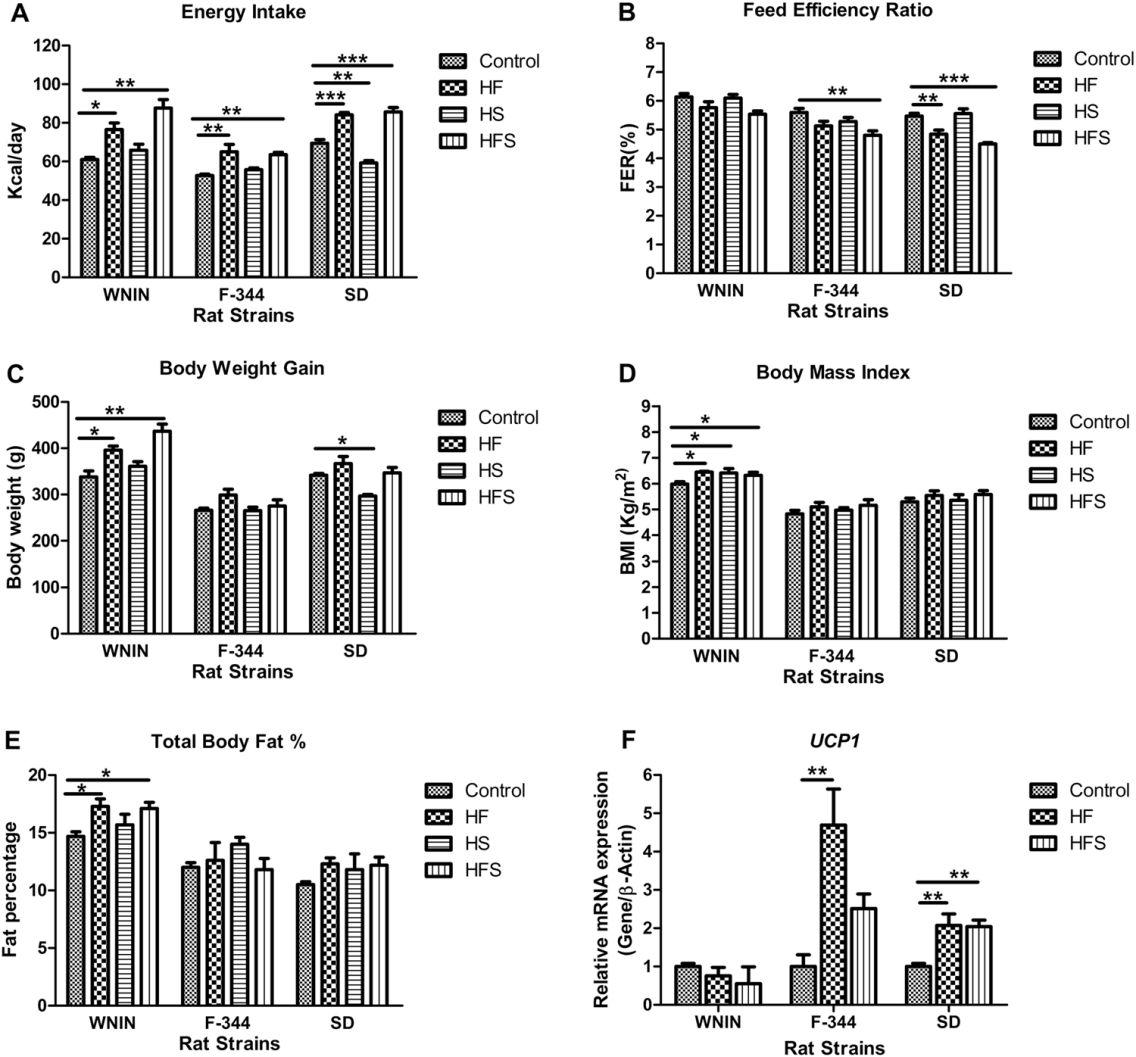
Effect of high calorie environment on feeding behavior, body composition and thermogenesis. Rats (WNIN, F-344 and SD) were fed with control, HF, HS, and HFS diets for 13 weeks and assessed (**A**) Mean energy intake (Kcal/day). (**B**) Feed efficiency ratio (FER). (**C**) Body weight gain. (**D**) Body mass index (BMI). (**E**) Total body fat percent. (**F**) Relative mRNA expression of UCP1 in brown adipose tissue. Data was presented as mean ± SEM (n = 6 per group). Diets: control; HF, high fat; HS, high sucrose; HFS, high fat sucrose. UCP1, uncoupling protein 1. *P < 0.05; **P < 0.01; ***P < 0.001 statistically significance compared to their respective controls. Groups were compared using one way ANOVA.

To examine the role of genetic predisposition in different genetic backgrounds, rats (WNIN, F-344 and SD) were fed with high calorie diets for 13 weeks: high fat (HF), high sucrose (HS) and high fat sucrose (HFS) groups, hereafter diet groups were defined as HF, HS & HFS and compared with their controls respectively. Differential food intake response was noticed among three genetically different rat strains when subjected to high calorie diets. All the three rat strains showed increased energy consumption (Kcal/day) in HF& HFS significantly (p < 0.001), whereas, in WNIN & F-344 with HS, energy consumption was comparable and in SD it was decreased significantly (p < 0.01) (Fig. 1A). In WNIN and F-344, feed efficiency ratio (FER) was comparable in HF, but in SD it was decreased significantly (p < 0.01), whereas in HS were comparable among all three strains. F-344 and SD showed significantly (p < 0.001) decreased FER in HFS and in WNIN it was comparable (Fig. 1B). In WNIN, increased body weight gain was observed significantly (p < 0.05) in HF&HFS and was comparable in F-344 and SD. Whereas, in WNIN & F-344 with HS, body weight was comparable and in SD it was decreased significantly (p < 0.05) (Fig. 1C).

In WNIN, body mass index (BMI) was significantly (p < 0.05) increased in all the diet groups, similarly, the total body fat percent (fat %) was increased significantly (p < 0.05) in HF & HFS but was comparable with HS. In F-344 and SD, the BMI and fat percent were observed comparable in all diet groups (Fig. 1D,E). The lean body mass and fat free mass percent (LBM% & FFM%) were comparable in all the diet groups and rat strains studied (Supplementary Table S2). The gene expression of uncoupling protein-1(UCP-1) in brown adipose tissue (BAT), which determines the energy expenditure by non-shivering thermogenesis was significantly (p < 0.01) up regulated in F-344 and SD among all diet groups, whereas in WNIN the levels were comparable (Fig. 1F).

### Visceral adiposity and adipocyte histology

**Figure 2:**
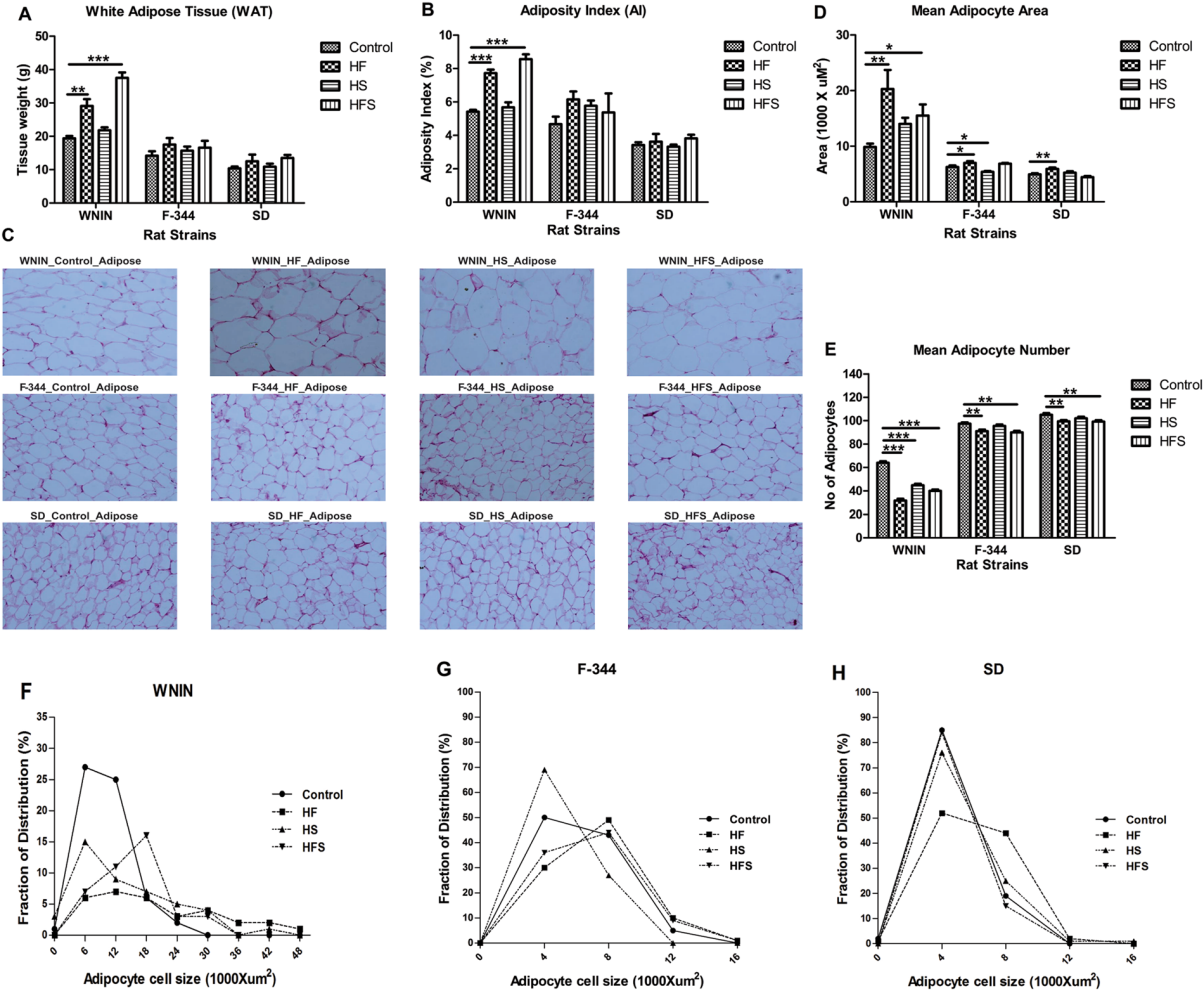
Effect of high calorie environment towards visceral adiposity and adipocyte histology in genetically different rat strains. After the completion of feeding experiment, rats (WNIN, F-344 and SD) were sacrificed and assessed visceral adiposity and adipocyte histology. (**A**) Dissection weights of white adipose tissue (WAT). (**B**) Adiposity index was calculated to measure central adiposity. (**C**) Hematoxylin-Eosin (H&E) staining was performed to determine the morphology of retro adipose tissue. (**D** & **E**) Mean adipocyte area and adipocyte number was calculated in retro adipose tissue. (**F**–**H**) Fraction of distribution of adipocytes in retroadipose tissue. Diets: control; HF, high fat; HS, high sucrose; HFS, high fat sucrose. *P < 0.05; **P < 0.01; ***P < 0.001 statistically significance compared to their respective controls s. Groups were compared using one way ANOVA.

To examine whether increased body weight gain was associated with visceral adiposity; we measured white adipose tissue (WAT) weights, calculated adiposity index and adipocyte hypertrophy among WNIN, F-344 and SD rat strains. In WNIN, WAT levels as well as adiposity index (AI) were observed to be significantly (p < 0.001) higher in HF & HFS, whereas in F-344 and SD were comparable among all the diet groups (Fig. 2A,B). Further, in WNIN the individual fat types such as retroperitoneal, mesenteric, and epididymal; the ratios of retro & mesenteric fat to body weight were found to be significantly (p < 0.01) higher in HF & HFS. Whereas, in F-344 and SD they were comparable among all diet groups (Supplementary Table S3, Supplementary Fig. S1).

In WNIN, visceral adipose tissue showed increased hypertrophy with higher mean adipocyte area (100 cells/ animal, 4 animals/group) and decreased hyperplasia (low adipocyte number) significantly (p < 0.001) in HF & HFS, but the HS showed comparable mean adipocyte area (Fig. 2C–E). Further, considerable differences were observed in WNIN towards adipocyte cell size and distribution where the percent of small cell fraction distribution were found to be lower in all diet groups (Fig. 2F). In F-344 and SD, even though the HF showed significantly (p < 0.05) higher mean adipocyte area, it was relatively lower than WNIN. Similarly, F-344 and SD fed with HF, HFS showed significant (p < 0.01) increase in adipocyte number and the levels were relatively higher compared to WNIN. Further, the percent of adipocyte cell fraction distribution were comparable between F-344 and SD rats (Fig. 2D,E,G & H).

### Increased adiposity in WNIN is associated with progression of pro-inflammatory status

**Figure 3:**
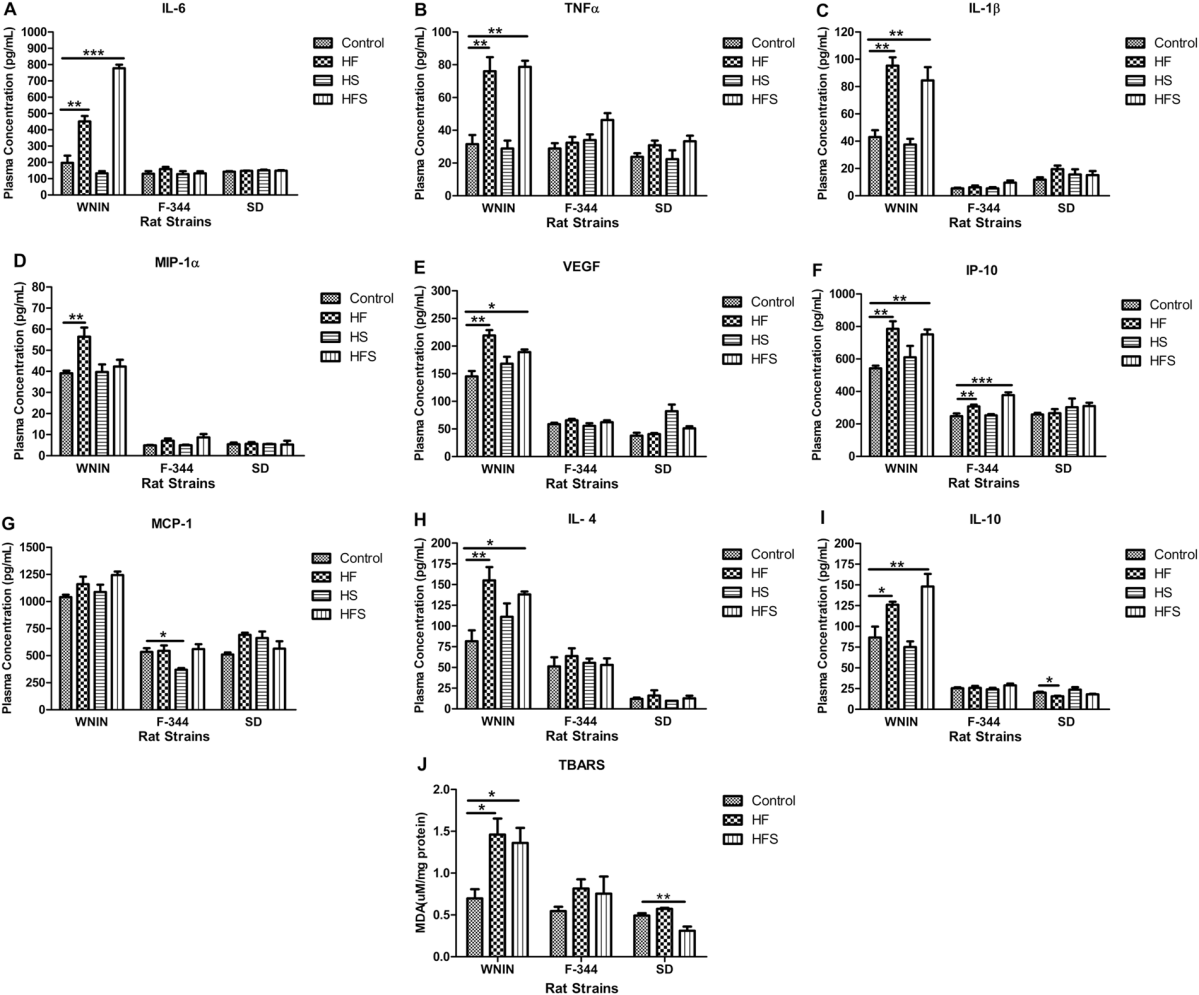
Effect of high calorie environment towards oxidative stress and pro-inflammatory status in rat strains. After the completion of feeding experiment, blood was collected; plasma was separated and estimated the levels of pro-inflammatory cytokines. (**A**) Interleukin 6; IL-6. (**B**) Tumor necrosis factor alpha; TNFα. (**C**) Interleukin 1 beta; IL-1β. (**D**) Macrophage inflammatory protein 1 alpha; MIP-1α. (**E**) Vascular endothelial growth factor; VEGF. (**F**) IFN gamma inducible protein 10; IP-10. (**G**) Monocyte chemotactic protein 1; MCP1 and anti -inflammatory cytokines such as (**H**) Interleukin 4; IL-4 and (**I**) Interleukin 10; IL-10. (**J**) TBARS assay was performed on liver tissue lysate to study oxidative stress. Data was presented as mean ± SEM (n = 6 per group). Diets: control; HF, high fat; HS, high sucrose; HFS, high fat sucrose. TBARS, Thio Barbituricacid Reactive Substances. *P < 0.05; **P < 0.01; ***P < 0.001 statistically significance compared to their respective controls. Groups were compared using one way ANOVA.

To investigate the relationship between increased adiposity and progression of pro-inflammatory status, we examined the potential role of liver oxidative stress in the development of inflammation by performing TBARS (Thiobarbituric Acid Reactive Substances) assay in liver tissue lysate. In WNIN, the hepatic MDA (Malondialdehyde) levels were significantly (p < 0.05) elevated in HF& HFS, whereas in F-344 it was comparable among all diet groups. On the other hand in SD, the MDA levels were significantly (p < 0.05) decreased in HFS and comparable in HF (Fig. 3J).

Further, we measured the levels of pro-inflammatory adipocytokines both in circulation and in visceral adipose tissue. In WNIN, the basal levels of plasma pro-inflammatory cytokines such as interleukin 6 (IL-6), tumor necrosis factor alpha (TNFα), interleukin 1 beta (IL-1β), vascular endothelial growth factor (VEGF), IFN-γ inducible protein 10 (IP-10; CXCL 10) were significantly increased in HF & HFS (Fig. 3A–C,E and F) but monocyte chemotactic protein-1 (MCP-1; CCL2) was comparable among all diet groups (Fig. 3G). However, macrophage inflammatory protein (MIP-1α, CCL3) was significantly (p < 0.01) elevated only in HF but comparable in HS & HFS (Fig. 3D).

In F-344, the plasma pro-inflammatory cytokines such as IL-6, TNF-α, IL-1β, MIP-1α, and VEGF were comparable in all diet groups (Fig. 3). However, increased levels of IP-10 in HF & HFS and decreased levels of MCP-1 in HS were observed significantly (p < 0.05) in F-344(Fig. 3F & G). In SD, levels of IL-6, TNF-α, IL-1β, MIP-1α, VEGF, IP-10, and MCP-1 were comparable in all diet groups (Fig. 3).

In WNIN, the basal levels of plasma anti- inflammatory cytokines such as interleukin 4 (IL-4) and interleukin 10 (IL-10) were significantly (p < 0.05) increased in HF & HFS but comparable in HS. In F-344 and SD, levels of IL-4 and IL-10 were comparable in all diet groups. However, in SD, the HF showed significantly (p < 0.05) decreased IL-10 (Fig. 3H & I).

In WNIN, consistent with plasma levels, visceral adipose tissue lysate showed significantly (p < 0.01) increased levels of pro-inflammatory cytokines such as IL-6, TNF-α, MCP-1, and MIP-1α in HF & HFS. Whereas, in F-344 and SD, the levels were comparable among all diet groups (Table 1).

**Table 1:** Expression of pro-inflammatory cytokines in visceral adipose tissue.

Conc. (pg/mg)	WNIN			F-344			SD		
	Control	HF	HFS	Control	HF	HFS	Control	HF	HFS
**IL-6**	1.91 ± 0.25	4.42 ± 0.53 **	5.05 ± 0.308 **	2.22 ± 0.21	2.54 ± 0.205	2.06 ± 0.244	3.15 ± 0.135	3.45 ± 0.305	3.51 ± 0.117
**TNF-α**	1.4 ± 0.125	6.05 ± 0.612 **	5.75 ± 0.663 **	0.54 ± 0.058	0.773 ± 0.124	0.68 ± 0.147	0.863 ± 0.159	0.95 ± 0.121	1.11 ± 0.091
**MIP-1α**	0.91 ± 0.288	3.59 ± 0.655 **	4.25 ± 0.345 **	0.953 ± 0.06	1.19 ± 0.055	1.23 ± 0.096	1.13 ± 0.064	1.3 ± 0.181	1.21 ± 0.239
**MCP-1**	7.26 ± 0.796	13.8 ± 0.47 **	15.6 ± 1.36 **	5.14 ± 0.591	6.21 ± 0.325	5.48 ± 0.267	6.94 ± 0.288	7.93 ± 0.085	7.44 ± 0.5

Retroperitoneal fat was used for the preparation of total tissue lysate and estimated the protein levels of pro-inflammatory cytokines such as interleukin 6, IL-6; tumor necrosis factor alpha, TNFα; macrophage inflammatory protein 1 alpha, MIP-1α; monocyte chemotactic protein 1, MCP1 among WNIN, F-344 and SD rats under high calorie environment. Data was presented as mean ± SEM (n = 6 per group). Diets: control; HF, high fat; HS, high sucrose; HFS, high fat sucrose. *P < 0.05; **P < 0.01; ***P < 0.001 statistically significance compared to their respective controls. Groups were compared using one way ANOVA.

### Obesity associated inflammation impaired glucose homeostasis and insulin sensitivity in WNIN

**Figure 4:**
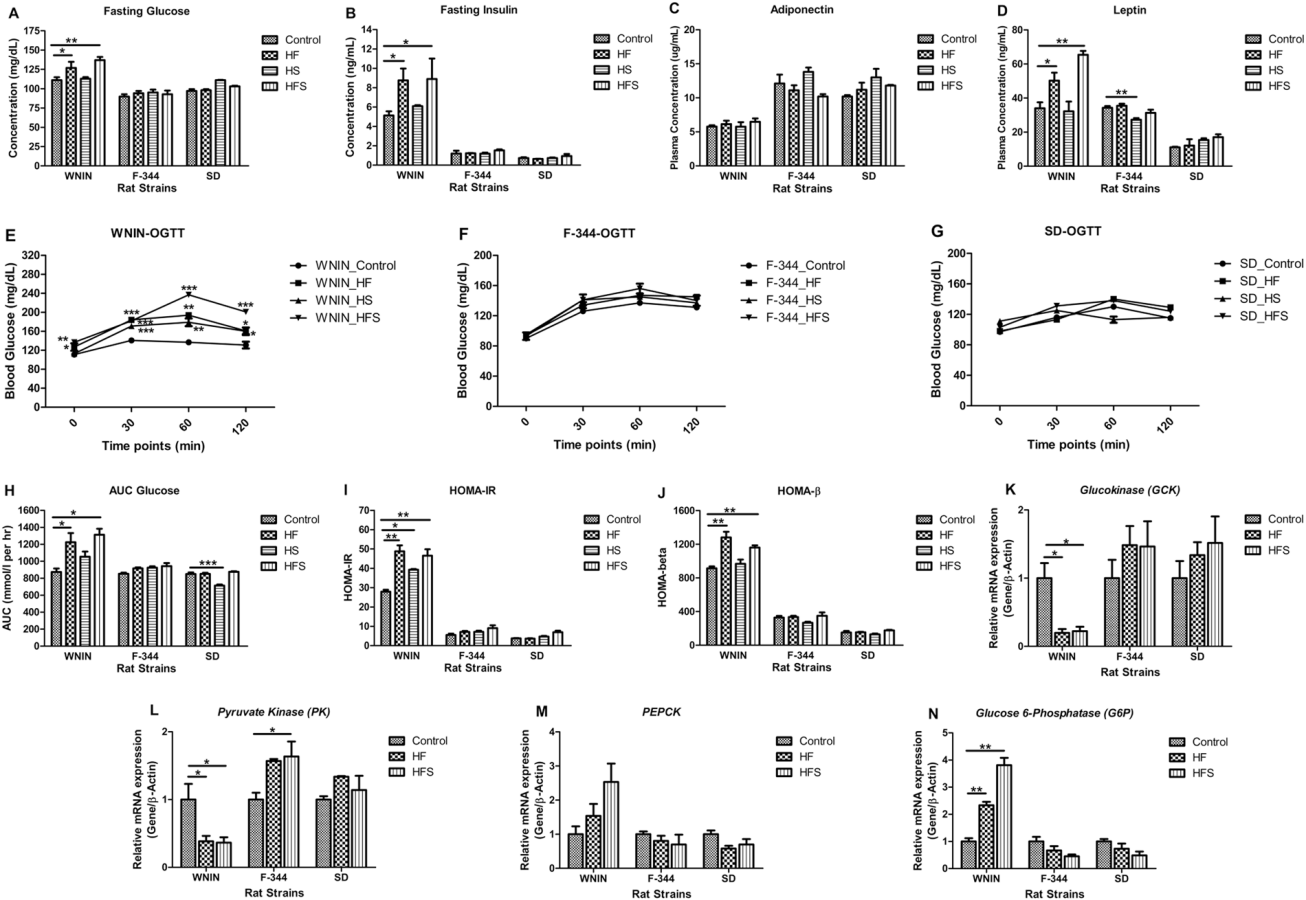
Effect of high calorie environment on glucose homeostasis: glucose tolerance, insulin sensitivity, glycolysis and gluconeogenesis in genetically different rat strains. Fasting plasma was used to estimate the concentrations of (**A**) Glucose. (**B**) Insulin. (**C**) Adiponectin. (**D**) Leptin. (**E**–**G**) OGTT was performed to assess glucose tolerance. (**H**) AUC Glucose was calculated based on the OGTT data. (**I** & **J**) HOMA-IR and HOMA-β was calculated to assess the insulin sensitivity. Relative mRNA expression of (**K**) glucokinase (GCK), (**L**) pyruvate kinase (PK), (**M**) Phosphoenolpyruvate carboxykinase (PEPCK), and (**N**) Glucose-6-phosphotase (G6Pase) to understand the regulation of glycolysis and gluconeogensis in liver. Data was presented as mean ± SEM (n = 6 per group). Diets: control; HF, high fat; HS, high sucrose; HFS, high fat sucrose; OGTT, Oral Glucose Tolerance Test; AUC Glucose, Area under curve for glucose; HOMA-IR, Homeostasis model assessment of insulin resistance; HOMA-β, Homeostasis model assessment of β-cell function. *P < 0.05; **P < 0.01; ***P < 0.001 statistically significance compared to their respective controls. Groups were compared using one way ANOVA.

In WNIN, fasting glucose and insulin levels were found to be significantly (p < 0.05) increased in HF & HFS and in HS it was comparable. Whereas, in F-344 and SD, the levels were comparable in all diet groups (Fig. 4A & B). Plasma adiponectin levels were comparable in all diet groups among the three rat strains. But amongst the strains, WNIN had the lowest expression (Fig. 4C). Similarly, in WNIN, the plasma leptin was significantly (p < 0.01) increased in HF & HFS but was comparable in HS. However, in F-344 the levels were significantly (p < 0.01) decreased in HS but was comparable in HF & HFS. In SD, the same was comparable in all diet groups (Fig. 4D).

Further, it was noticed that only WNIN showed significantly (p < 0.001) delayed glucose clearance from circulation but not the F-344 and SD when challenged with glucose orally for all diet groups (Fig. 4E–G). In line, the homeostasis model assessment of insulin resistance (HOMA-IR) score was significantly (p < 0.01) higher in WNIN. Further, the homeostasis model assessment of β-cell function (HOMA-β) and area under curve for glucose (AUC glucose) scores were significantly (p < 0.01) higher in HF & HFS but comparable for HS in WNIN (Fig. 4I,J & H). In F-344 and SD; HOMA-IR, HOMA-β and AUC glucose were found to be comparable in all diet groups (Fig. 4I,J & H).

In WNIN, qPCR mRNA expression studies in liver revealed that enzymes involved in glycolysis; glucokinase (GK) and pyruvate kinase (PK) were significantly (p < 0.05) down regulated in HF & HFS (Fig. 4K & L), whereas in F-344 and SD, the same were comparable (Fig. 4K & L). Further, gluconeogenesis was determined by mRNA expression of rate limiting enzymes i.e, PEPCK and G6Pase. In WNIN, gene expression of PEPCK was relatively higher though statistically not significant in HF & HFS. On the other hand, G6Pase expression was significantly (p < 0.01) upregulated (Fig. 4M & N). However, in F-344 and SD, the expression of PEPCK and G6Pase were found to be comparable in all diet groups (Fig. 4M & N).

### Obesity associated inflammation altered lipid metabolism in WNIN

**Figure 5:**
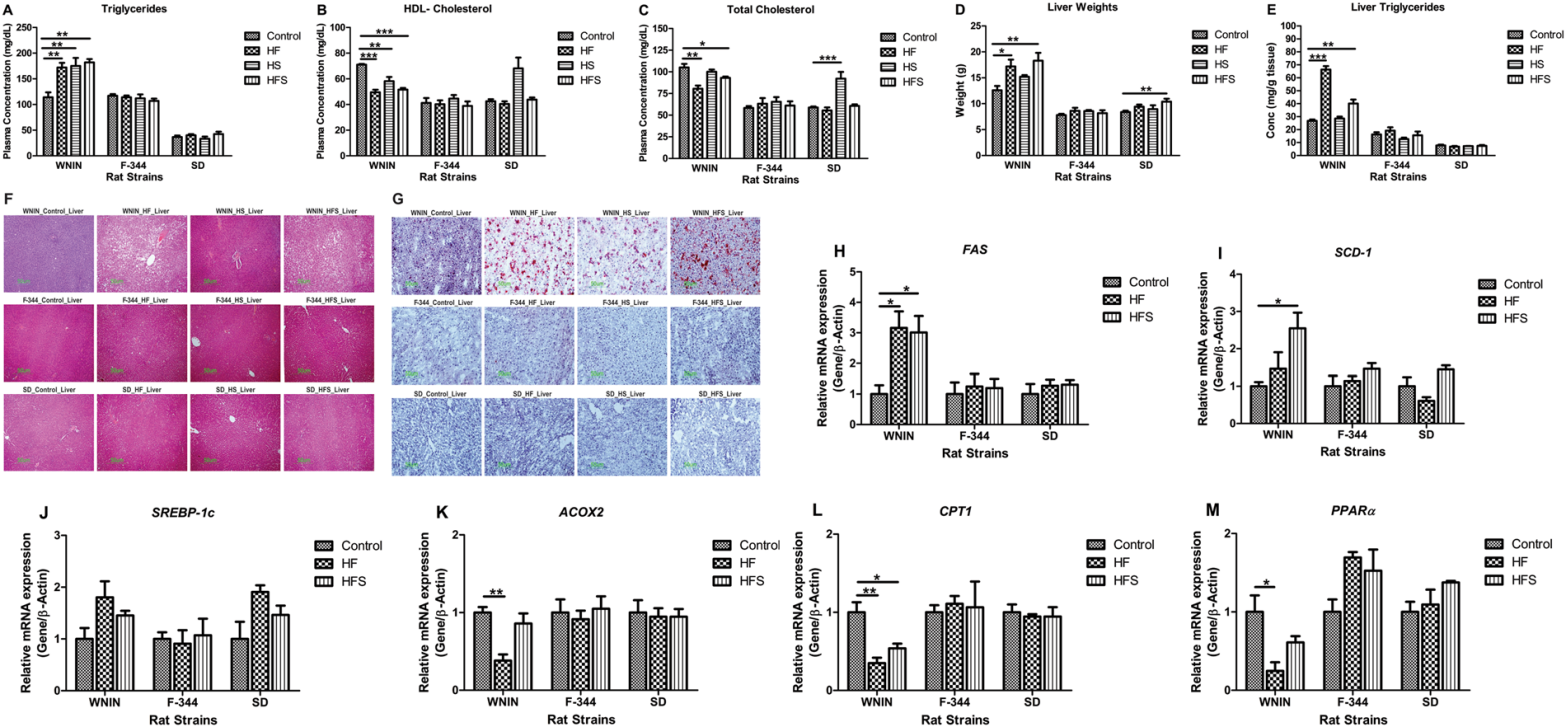
Effect of high calorie environment on lipid metabolism: Lipid profile, lipogenesis and beta oxidation in genetically different rat strains. Fasting plasma was used to estimate the concentrations of (**A**) Triglycerides. (**B**) HDL- Cholesterol. (**C**) Total - cholesterol. (**D**) Dissection weights of liver. (**E**) Liver triglycerides levels were measured to determine the fatty liver condition. (**F** & **G**) Hematoxylin-Eosin (H&E) stain and Oil Red O stain were performed on liver sections to assess hepatic lipid droplet storage. Relative mRNA expression of (**H**) Fatty acid synthase (FAS), (**I**) Stearoyl-CoA desaturase-1 (SCD-1), (**J**) Sterol regulatory element-binding protein 1-c (SREBP-1c), (**K**) Acyl-CoA Oxidase 2 (ACOX2), (**L**) Carnitine palmitoyltransferase 1 (CPT1), and (**M**) Peroxisome proliferator-activated receptor α (PPARα) to understand the regulation of hepatic lipogenesis and beta oxidation. Data was presented as mean ± SEM (n = 6 per group). Diets: control; HF, high fat; HS, high sucrose; HFS, high fat sucrose. *P < 0.05; **P < 0.01; ***P < 0.001 statistically significance compared to their respective controls. Groups were compared using one way ANOVA.

Plasma triglyceride levels were significantly (p < 0.01) elevated and HDL-cholesterol & Total- cholesterol levels were significantly (p < 0.001) decreased in all the diet groups of WNIN. On the other hand, in F-344 and SD, the same were comparable in all diet groups (Fig. 5A–C).

In WNIN, HF & HFS showed significantly (p < 0.001) higher liver weights; whereas in HS, it was comparable (Fig. 5D). In consistent with the above, HF & HFS of WNIN showed increased vacuolar structures, lipid droplet accumulation and significantly (p < 0.05) elevated levels of triglycerides in liver (Fig. 5E–G). In F-344 & SD, all diet groups showed no lipid droplet accumulation, comparable liver triglycerides and liver weights (Fig. 5D–G).

Then we investigated the regulation of hepatic lipid metabolism. It was observed that WNIN showed significantly (p < 0.05) elevated expression of lipogenic genes: FAS and SCD1 in HF & HFS (Fig. 5H & I). However, SREBP-1c; the transcriptional regulator of lipogenesis showed comparable expression (Fig. 5J). On the other hand, the genes involved in beta oxidation of lipids i.e. ACOX2, CPT1 and PPARα were significantly (p < 0.01) down regulated in HF & HFS of WNIN (Fig. 5K–M). However, in F-344 and SD, high calorie diet neither affected lipogenesis nor the beta oxidation of fatty acids as evidenced by comparable expression of all rate limiting genes involved in lipid metabolism (Fig. 5H–M).

### Comparative analysis of obesity characteristics among the strains

Obesity characteristics were compared among all diet groups and rat strains. Data was normalized against their respective control groups after obtaining statistical mean values for each parameter and measured the percentage change. It was noticed that the percent of increase for the studied parameters were higher in HF & HFS of WNIN (Table 2).

**Table 2:** Assessment of effect of individual high calorie diets towards obesity development in WNIN, F-344 and SD.

% of increase	High fat diet (HF)			High Sucrose diet (HS)			High fat sucrose diet (HFS)		
	WNIN	F-344	SD	WNIN	F-344	SD	WNIN	F-344	SD
Insulin	71	3	−12	19	−1	1	73	28	29
AUC Glucose	40	8	0	21	9	−16	50	11	3
Fasting Triglycerides	51	−3	9	54	−4	−9	60	−9	30
WAT	50	23	20	12	11	5	93	17	30
Retro fat	50	21	7	3	−1	−4	96	24	49
Plasma Leptin	48	3	9	−5	−20	39	92	−9	53
Plasma IL-6	129	22	3	−32	−2	6	295	2	4
Plasma TNF-α	141	12	30	−8	18	−6	150	60	40

The data was presented as the percentage change to their respective control groups. Data was normalized against to their respective control groups after obtaining statistical mean values for the each parameter and measured the percent of change by calculating {(experimental − control)/control}*100 and represented the values as increased or decreased.

## Discussion

Obesity is a pathological condition with excess body fat accumulation and the reasons for this modern malady could be due to genetic predisposition^14–16^ and/or calorie rich diet consumption^7, 8, 29^ or the interplay of both (diet-gene interactions)^30–32^. Several animal studies have earlier reported that the high calorie diet rich in fats and sugars can precipitate obesity and this was demonstrated on a single genetic back ground^33–35^. However, this would not answer the question of why certain individuals or groups develop obesity and others do not, while being fed the same calorie rich diet. Though earlier studies tried to address this paradox, the results remained largely inconclusive^24, 25, 36–38^. There is a need to explore about the choice of diet selection and probable nature of an individual’s genetic response to the development of obesity. Therefore, this study focused on the effect of different diets and their selective response on various genotypes of laboratory rats in the development of obesity. All in all we investigated the obesogenic potential of three rat strains with different genetic backgrounds (WNIN, F-344 and SD) by exposing them to a high calorie environment.

Among the three strains studied, WNIN showed significantly increased energy consumption and decreased energy expenditure in HF and HFS diets, resulting in increased body weight gain, BMI, fat%, WAT weights, and AI; the hallmarks of central obesity (Figs 1 and 2). However, the feed efficiency ratio (FER) which is a reflection of feed utilization vis-a-vis weight gain was found to be comparable in all the dietary groups, suggesting that more than higher energy consumption, it is the type of macronutrients and its combination, which are more important in precipitation of obesity as shown earlier^2–5^. The other two strains, F-344 and SD rats under the same high calorie environment, did not show any tendency for obesity, despite having increased energy consumption. This is actually a reflection of their metabolic profiles and they being generally more active^27^. Studies reported that the impaired body weight gain and central adiposity are due to enhanced basal metabolism through increased energy expenditure by acceleration of fatty acid oxidation & thermogenesis^39, 40^. In the present study our results confirmed that in WNIN, high calorie diets modulated the regulation of hepatic lipid metabolism by promoting increased hepatic lipogenesis and decreased fatty acid oxidation significantly (Fig. 5). The significant increase of thermogenesis and fatty acid oxidation in HF & HFS diets of F-344 and SD suggest that even a high calorie environment did not alter the energy metabolism (Figs 1 and 5), thus protecting them from developing obesity. In short, though all the three strains were exposed to high calorie diets, only WNIN showed its vulnerability to develop obesity, showing that certain genotypes are more prone than others. To such a strain (genotype) an exposure to high calorie diet is an additional environmental trigger in the development of metabolic disorder.

Under the influence of high calorie environment, WNIN developed increased adipocyte hypertrophy, visceral adiposity and hepatic oxidative stress (Figs 2 and 3), which in turn stimulated the chronic low grade inflammation, as shown by the elevation of pro- inflammatory adipocytokines both at systemic and tissue level (Fig. 3 & Table 1). The increased levels of anti- inflammatory cytokines such as IL-4 and IL-10 in WNIN could explain the possible underlying mechanism to counteract the detrimental effects of inflammation. Research on high fat diet induced obesity demonstrated that visceral adiposity and associated inflammation play an important role in the development and progression of metabolic syndrome^11–13^. Studies have shown that adiponectin & leptin exhibit anti diabetic, anti atherogenic and anti inflammatory properties; involved in the regulation of glucose and lipid metabolism by enhancing insulin sensitivity and protects against chronic inflammation^41–44^. In WNIN, hyperleptinemia and hypoadiponectinemia (Fig. 4) condition under high calorie environment indicate its limitations in energy expenditure and glucose utilization; which inturn led to the promotion of increased hepatic gluconeogenesis, decreased glycolysis and fatty acid oxidation (Figs 4 and 5). Further, the increased visceral adiposity and the associated inflammation led to insulin resistance and impaired glucose tolerance (IGT). The decreased adiponectin: leptin ratio, higher scores for HOMA-IR, HOMA-β and AUC glucose corroborate beta cell dysfunction and altered glucose homeostasis (Fig. 4 & Supplementary Fig. S2)^45, 46^ in WNIN. In addition, increased plasma triglycerides, decreased HDL-cholesterol & HDL-C/total-C ratio, deposition of hepatic lipid droplets resulted in dyslipidemia and fatty liver condition which was observed only in WNIN. (Fig. 5 & Supplementary Fig. S3). This was in concurrence with earlier reports that have shown, diet rich in high saturated fats and sugars modulates the ratio of adiponectin:leptin & HDL-C/Total-C resulting in altered glucose & lipid metabolism and development of obesity associated co-morbidities^47, 48^. The study thus clearly showed the vulnerability of WNIN to develop obesity, which may be due to genotype-diet interaction by epigenetic mechanisms. The comparative analysis among the strains and high calorie diets showed that the diet rich in saturated fats alone or in combination with sucrose have discerning influence on the development of obesity only in WNIN. Further to mention, the diet in combination with saturated fat & sucrose (HFS) was shown to have more detrimental effect than the saturated fat alone and this could be due to the synergistic effect of diet composition as well as the genetic predisposition, as evident in WNIN (Table 2).

The study results indicate that certain genotypes undergo epigenetic modifications at protein and or gene promoter levels when interact with nutritional factors. This could be one of the potentially important contributors for inter-individual differences in fat deposition and obesity. We have carried out whole transcriptome analysis of liver and adipose tissues of these animals and it has revealed very interesting results that should explain the obesogenic tendency of WNIN rats (unpublished data). As mentioned earlier, two obese rat strains, i.e., WNIN/Ob and WNIN/GR-Ob, spontaneously arose from WNIN stocks, maintained at the Centre and these are well characterized and used for several studies on obesity and associated metabolic disorders^28, 49–52^. Knowing the obesogenic tendency of the WNIN strain as revealed in this study, it is no wonder that the obese mutant strains mentioned above evolved from this parental stock.

The effect of HS diet, which did not aid in obesogenesity in WNIN, which also did not alter many of the parameters studied in these three strains, is worth studying further in the area of diet - gene interaction. It is to be noted that the HS diet group of WNIN did develop insulin resistance and dyslipidemia but did not result in increased body weight gain and visceral adiposity. Several studies have earlier demonstrated that feeding of male Wistar rats with high sucrose did not induce obesity through reduced energy expenditure but was shown to cause impairment of insulin action and elevation of plasma triglycerides^53–55^. The importance of genotype in developing a metabolic disorder was evident again, as F-344 and SD did not show any abnormalities with HS diet.

Further, molecular analysis of various signaling pathways involved in energy regulation, especially glucose and lipid homeostasis could yield much more interesting results. This in turn could lead to identification and characterization of genes/proteins which are either susceptible or resistant to diet induced obesity. To the best of our knowledge this is the first study of its kind, wherein the effect of high calorie diets, rich in fat or sucrose or in combination of both in different genetic backgrounds was attempted to understand the interplay in the manifestation of metabolic disease. The study concludes that genotype determines one’s response to an environmental variable like diet leading to susceptibility or resistance in the development of obesity and associated co-morbidities.

## Materials and Methods

### Animal Experiment

The study was conducted after obtaining ethical approval from the Institutional Animal Ethical Committee (IAEC: P30/7-2011/NVG), National Centre for Laboratory Animal Sciences (NCLAS), National Institute of Nutrition (NIN), Hyderabad, India. All the methods were performed at NCLAS in accordance with the relevant guidelines and regulations of the Committee for the Purpose of Control and Supervision of Experimentation on Animals (CPCSEA, India). Strains of male Wistar/NIN (WNIN), Fischer-344/NIN (F-344) and Sprague Dawley/NIN (SD) weaning rats (n = 6) having an average weight of 30 g–35 g obtained from NCLAS, NIN and were used for the study. The animals were housed individually in sterilized wire mesh bottomed polypropylene cages and maintained under standard lighting conditions (12-hour light/ dark cycle). Temperature and relative humidity were kept constant at 22 ± 2 °C and 50 ± 5% respectively.

### Diet

Control diet is prepared according to the AIN-93G formulation^56^ which has total energy of 395 Kcal/100 g (64% carbohydrates, 20% proteins and 16% fat); high fat diet (HF) has total energy of 540 Kcal/100 g (20% carbohydrates, 20% proteins and 60% fat); high sucrose diet (HS) has total energy of 395 Kcal/100 g (64% carbohydrates, 20% proteins and 16% fat) and high fat sucrose diet (HFS) has total energy of 480 Kcal/100 g (35% carbohydrates, 20% proteins and 45% fat) contributing from various macronutrients. The amount of fiber, mineral elements, and vitamins were maintained according to standard diet formulation. All the diets were prepared using local ingredients present in the market (Supplementary Table S1).

### Study Design

Each strain of WNIN, F-344, and SD were divided into 4 groups (n = 6) such as control, HF, HS & HFS and were fed with respective diets. Animals were fed adlibitum over a period of 13 weeks and had free access to deionized water throughout the experiment. Food intake was measured for everyday and body weights were recorded for every week. At the end of the experiment, animals were sacrificed and tissues were dissected out, snap frozen in liquid nitrogen immediately and stored at −80 °C until further analysis.

### Anthropometric Measurements

Energy intake, FER and BMI were measured as described previously^57^. Energy Intake (Kcal/day) = mean food consumption x dietary metabolized energy; Feed Efficiency Ratio (FER) = (mean body weight gain × 100)/energy intake; Body Mass Index (BMI) = Body weight (Kg)/length (m^2^).

### Body Composition

The body composition of the animals was determined at the end of the experiment by TOBEC (Total Body Electrical Conductivity) using small animal body composition analysis system (EM-SCAN, Model SA-3000 Multi detector, Springfield, USA). Lean body mass, fat free mass and total body fat percent were measured as described previously^58^.

### Adiposity Index (AI)

AI was calculated as described previously^59^. AI = (WAT weight × 100)/(Body weight − WAT weight). WAT weight includes retroperitoneal fat, mesenteric fat, epididymal fat, and omental fat.

### Plasma Parameters

Plasma insulin & adiponectin was measured by sandwich ELISA method according to manufacturer’s instructions and kits were obtained from Millipore, USA & M/S Mediagnost, Germany respectively. Plasma glucose, triglycerides, total cholesterol and HDL-cholesterol levels were measured by using commercially available enzyme-based assay kits (Biosystems S.A, Spain) according to manufacturer’s instructions.

### Oral Glucose Tolerance Test (OGTT)

OGTT was performed to measure the glucose tolerance. After an overnight fasting, dextrose solution (40% wt/vol) was administered intra-gastrically into rat at a dose of 2.5 g/kg body weight and blood samples were collected from supra orbital under mild Isoflurane anesthesia at 0, 30, 60 and 120 min time points. Glucose levels were measured at all time points. Glucose tolerance was measured during OGTT by calculating area under curve (AUC) for glucose by trapezoidal method^60^.

### Insulin Resistance

Homeostasis model assessment of insulin resistance (HOMA-IR) and homeostasis model assessment of β-cell function (HOMA-β) were calculated from fasting glucose and insulin values using the following formulae as described previously^45, 46^ to assess insulin resistance. HOMA-IR = [fasting glucose (mmol/L) × fasting insulin (uIU/mL)]/22.5; HOMA-β = [20 × fasting insulin (uIU/mL)]/[fasting glucose (mmol/L) − 3.5].

### Rat Cytokine/Chemokine Profile

The adipocytokines such as leptin, macrophage inflammatory protein-1 alpha (MIP-1α, CCL3), interleukin-4 (IL-4), interleukin-1 beta (IL-1β), interleukin-6 (IL-6), interleukin-10 (IL-10), macrophage chemo attractant protein-1 (MCP-1, CCL2), IFN-gamma-inducible protein 10 (IP*-*10, CXCL10), vascular endothelial growth factor (VEGF), and tumor necrosis factor-alpha (TNF-α) were estimated both in circulation as well as in visceral adipose tissue by Milliplex Rat cytokine immunoassay kit (Millipore, USA) according to manufacturer’s instructions.

### Determination of Liver Triglycerides

Liver triglyceride levels were measured as described previously^61^. For hepatic triglyceride content, the value was normalized to tissue weight and expressed as mg/g of liver.

### TBARS Assay

Quantitative determination of Thiobarbituric Acid Reactive Substances (TBARS) were assessed in rat liver tissue lysate by calorimetric assay procedure using commercially available kits (BioAssay Systems, USA) according to manufacturer’s instructions.

### Histopathology

Liver and visceral adipose tissue samples were stored in 10% neutral buffered formalin solution immediately after dissection, embedded in paraffin and 4 µ sections were stained with hematoxylin and eosin (H&E). Specimens were examined by microscopy (Nikon-Eclipse E800 microscope, Nikon Corporation, Tokyo, Japan) at 10× and 20× magnification and analyzed with Image-Pro Plus software (Media cybernetics, Bethesda, USA). H&E of liver was assessed for extensive vacuolar structures and fresh frozen liver tissue samples were processed for Oil Red O staining to confirm for neutral lipid accumulation. H&E adipose was used to study mean adipocyte area & adipocyte cell number and was calculated by number of cells/15 sq.mm, 100 cells/animal in 4 animals per group.

### Gene Expression Studies

Liver and brown adipose tissue (150 mg) was homogenized in trizol (Sigma-Aldrich,Co. USA) for RNA extraction. RNA purity and integrity was measured using Nanodrop 2000 spectrophotometer (Thermo Scientific, USA) and agarose gel electrophoresis. cDNA was synthesized by reverse transcription from 2 ug of total RNA using cDNA synthesis kit (Thermo Fischer Scientific Inc.). Real time quantitative PCR (CFX 96, Biorad, USA) was carried out by three steps PCR with melt curve method using SYBR Premix Ex Taq kit (Takara Bio Inc.). Primers were obtained from Sigma-Aldrich and the sequence were: AAGGTGCCCCTCTATCTGGAA (forward) and AAATATCCCCCAGAGCAAGGTG (reverse) for Stearoyl-CoA desaturase-1 (SCD 1); CCACCGCCTACTACTCCTTA (forward) and CTGCTCAAACGATGTGTCTC (reverse) for Fatty acid synthase (FAS); CGCTACCGTTCCTCTATCAATGAC (forward) and AGTTTCTGGTTGCTGTGCTGTAAG (reverse) for Sterol regulatory element-binding protein-1c (SREBP-1c); GCAGCTCGCACATTACAAGG (forward) and CTCTGCTCTGCCGTTGACTT (reverse) for Carnitine palmitoyltransferase I (CPT1); CTTGATCCGGAAGGATGCCA (forward) and TGCTTCTCGGTCCCAAATCC (reverse) for Acyl-CoA Oxidase 2(ACOX2); TCCTCTGGTTGTCCCCTTGA (forward) and CAGTCTTGGCTCGCCTCTAA (reverse) for Peroxisome proliferator-activated receptor alpha (PPAR-α); CAGTGGAGCGTGAAGACAAA (forward) and CTTGGTCCAATTGAGGAGGA (reverse) for Glucokinase (GCK); CCAAGGGACTGAGATACGA (forward) and AGGTCCACCTCAGTGTTTGG (reverse) for Pyruvate kinase (PK); AGCTCCGTGCCTCTGATAAA (forward) and AAAGTGAGCCGCAAGGTAGA (reverse) for Glucose-6-phosphatase (G6Pase); CCCAGGAGTCACCATCACTT (forward) and TTCGTAGACAAGGGGGACAC (reverse) for phosphoenolpyruvate carboxykinase (PEPCK); GACATCATCACCTTCCCGCT (forward) and CCGTATCGTAGAGGCCAATCC (reverse) for Uncoupling protein 1 (UCP1); and CCTTCCTGGGTATGGAATCCTG (forward) and GCTAGGAGCCAGGGCAGTAA (reverse) for β-Actin. Realtive mRNA expression for each gene was calculated using 2^−ΔΔCT^ method and normalized to β-Actin.

### Statistical Analysis

Data was expressed as mean ± standard error of mean (SEM). One way ANOVA and post-hoc test were performed to assess the significant differences among the groups. For parameters, where heterogeneity of the variance was significant, differences between groups were tested with non-parametric kruskal Wallis test. SPSS software (SPSS for Windows, Version 16.0., Chicago, SPSS Inc) and GraphPad Prism 5 was used for the statistical analysis. Differences with p-value < 0.05 were considered significant. *Represents statistical significance p < 0.05; ** represents statistical significance p < 0.01; ***represents statistical significance p < 0.001.

## Electronic supplementary material

Supplementary Information
