# Supplementary material for: Differential response of rat strains to obesogenic diets underlines the importance of genetic makeup of an individual towards obesity
Source: Sci Rep. 2017 Aug 22;7:9162. doi: 10.1038/s41598-017-09149-6 (PMC5567335; doi:10.1038/s41598-017-09149-6)
Supplement: Supplementary file 1 — Supplementary Information [file 41598_2017_9149_MOESM1_ESM.pdf]

Differential response of rat strains to obesogenic diets underlines the importance of genetic makeup of an individual towards obesity.

Naga Muralidhar M, SMVK Prasad, Kiran Kumar Battula, Giridharan N V and Rajender Rao Kalashikam*

National Centre for Laboratory Animal Sciences, National Institute of Nutrition, Hyderabad-500007, Telangana, India

*Corresponding author

e-mail: rkrajender@yahoo.com

Supplementary Table S1. Control and high calorie diet composition and energy levels.

Ingredient (g/100g)	Control (C)	High Fat (HF)	High Sucrose (HS)	High fat sucrose (HFS)
Corn Starch	63	27	---	42
Sucrose	---	---	63	---
Casein	20	27	20	24
Soybean Oil	7	---	7	---
Lard	---	36	---	24
Cellulose	5	5	5	5
Mineral Mixture (93G)	3.5	3.5	3.5	3.5
Vitamin Mixture (93G)	1.0	1.0	1.0	1.0
L- Cysteine	0.3	0.3	0.3	0.3
Choline bitartrate	0.25	0.25	0.25	0.25
Energy (Kcal/g)	3.95	5.4	3.95	4.8
Carbohydrate ratio (%)	64	20	64	35
Protein ratio (%)	20	20	20	20
Fat ratio (%)	16	60	16	45

Energy levels of the diets: Control diet, 3.95Kcal/g; High fat (HF) diet, 5.4 Kcal/g; High Sucrose (HS) diet, 3.95Kcal/g; High Fat Sucrose (HFS) diet, 4.8 Kcal/g.

Supplementary Table S2. Body composition of WNIN, F-344 and SD rats under high calorie environment.

	**WNIN**				**F-344**				**SD**			
Parameter	**Control**	**HF**	**HS**	**HFS**	**Control**	**HF**	**HS**	**HFS**	**Control**	**HF**	**HS**	**HFS**
**Lean body mass %**	84.4 ± 0.556	85.5 ± 0.390	83.0 ± 0.945	82.8 ± 0.539	83.9 ± 0.407	82.2 ± 0.647	83.5 ± 0.384	83.3 ± 1.05	89.8 ± 0.508	88.6 ± 0.707	88.1 ± 1.38	88.2 ± 0.776
**Fat free mass %**	43.5 ± 0.445	44.4 ± 0.312	42.3 ± 0.756	42.2 ± 0.431	48.7 ± 0.358	47.0 ± 0.790	48.2 ± 0.413	48.3 ± 1.07	52.3 ± 0.441	51.1 ± 0.714	51.4 ± 1.26	51.7 ± 0.626

Lean body mass and fat free mass percent (LBM% & FFM %) were assessed among WNIN, F-344 and SD under high calorie environment. Diets: control; HF, high fat; HS, high sucrose; HFS, high fat sucrose. Data was presented as mean ± SEM (n=6). Groups were compared using one way ANOVA.

Supplementary Table S3. Individual fat pad weights of White Adipose Tissue (WAT) in WNIN, F-344 and SD under high calorie environment.

	WNIN				F-344				SD			
Parameter	**Control**	**HF**	**HS**	**HFS**	**Control**	**HF**	**HS**	**HFS**	**Control**	**HF**	**HS**	**HFS**
**Retroperitoneal fat (g)**	10.9 ± 0.186	16.3 ± 1.75*	11.2 ± 0.548	21.4 ± 2.02***	7.58 ± 0.523	9.14 ± 0.985	7.52 ± 0.571	9.38 ± 1.58	3.70 ± 0.251	3.96 ± 0.185	3.56 ± 0.581	5.50 ± 0.650*
**Mesenteric fat (g)**	3.0 ± 0.225	4.5 ± 0.389**	3.52 ± 0.165	4.88 ± 0.233**	2.48 ± 0.289	2.77 ± 0.294	2.30 ± 0.372	2.85 ± 0.231	1.44 ± 0.172	1.62 ± 0.170	1.30 ± 0.100	1.94 ± 0.186
**Epididymal fat (g)**	4.87 ± 0.233	7.0 ± 0.293*	6.1 ± 0.382	9.1 ± 0.664***	6.68 ± 0.539	7.96 ± 0.955	6.38 ± 0.441	7.45 ± 1.17	4.46 ± 0.145	5.25 ± 0.904	4.15 ± 0.629	5.12 ± 0.460

Measurements of white adipose tissue (WAT); retroperitoneal fat, mesenteric fat and epididymal fat. Data was presented as mean ± SEM (n=6). Diets: control; HF, high fat; HS, high sucrose; HFS, high fat sucrose. *P<0.05; **P<0.01; ***P<0.001 statistically significance compared to their respective controls. Groups were compared using one way ANOVA.

Supplementary Fig. S1. Individual fat pads weight/body weight ratio

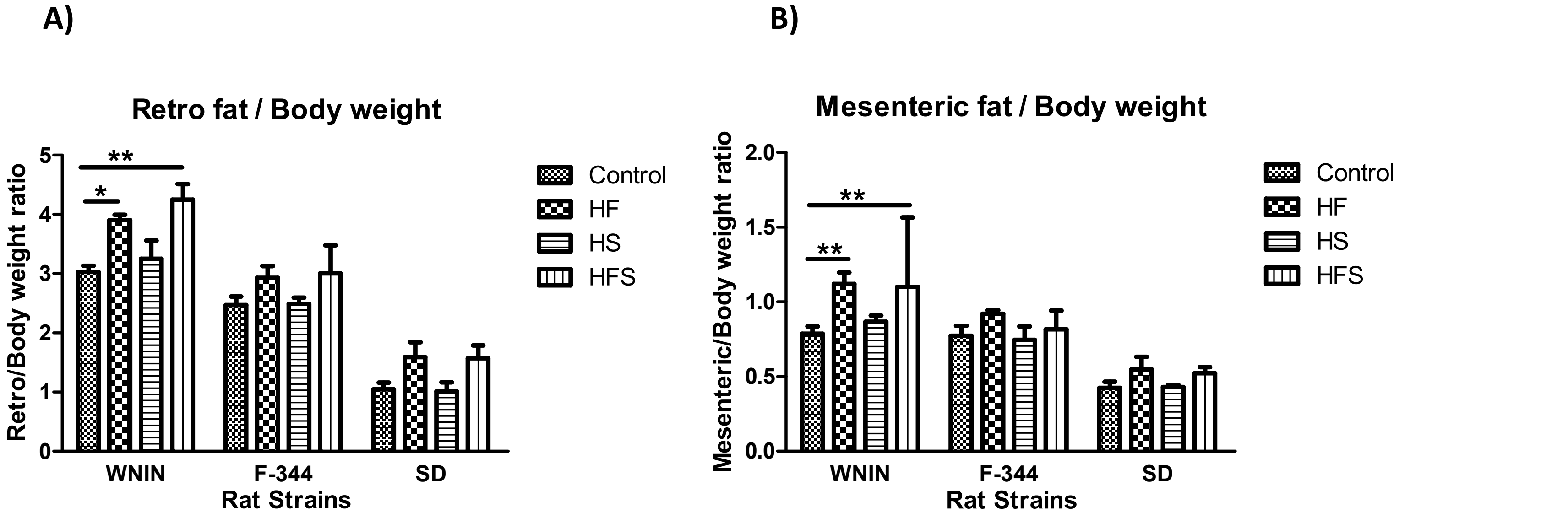

The ratios of (A) retroperitoneal fat to body weight and (B) mesenteric fat to body weight under high calorie environment. Data was presented as mean ± SEM (n=6). Diets: control; HF, high fat; HS, high sucrose; HFS, high fat sucrose. *P<0.05; **P<0.01 statistically significance compared to their respective controls. Groups were compared using one way ANOVA.

Supplementary Fig. S2. Adiponectin to Leptin & HDL-cholesterol to Total -cholesterol ratio

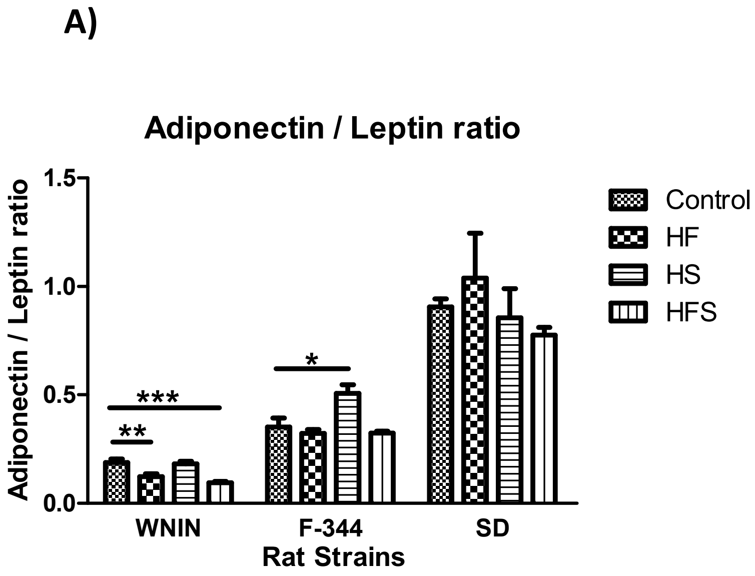

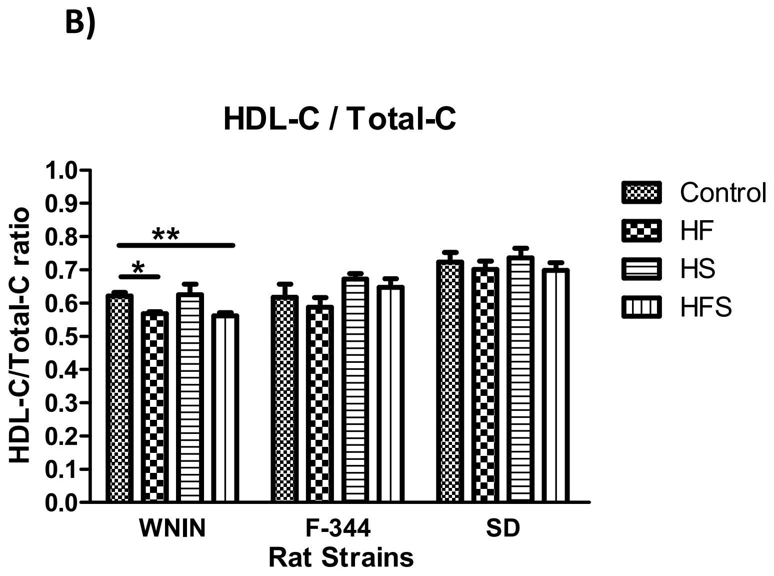

The ratios of (A) Adiponectin/Leptin and (B) HDL-cholesterol/Total-cholesterol under high calorie environment. Data was presented as mean ± SEM (n=6). Diets: control; HF, high fat; HS, high sucrose; HFS, high fat sucrose. *P<0.05; **P<0.01; ***P<0.001 statistically significance compared to their respective controls. Groups were compared using one way ANOVA.
